# Cloning, expression, and characterization of a recombinant xylanase from *Bacillus sonorensis* T6

**DOI:** 10.1371/journal.pone.0265647

**Published:** 2022-03-17

**Authors:** Assel Kiribayeva, Birzhan Mukanov, Dmitriy Silayev, Zhiger Akishev, Yerlan Ramankulov, Bekbolat Khassenov

**Affiliations:** 1 National Center for Biotechnology, Nur-Sultan, Kazakhstan; 2 L.N. Gumilyov Eurasian National University, Nur-Sultan, Kazakhstan; Konkuk University, REPUBLIC OF KOREA

## Abstract

Xylanase is one of industrial enzymes with diverse applications including the paper-bleaching industry and feed additives. Here, a strain having xylanolytic activity and identified as *Bacillus sonorensis* T6 was isolated from soil. A secretory enzyme was identified by mass-spectrometry as a xylanase of glycosyl hydrolase family 11, with a molecular weight of 23.3 kDa. The xylanase gene of *Bacillus sonorensis* T6 was cloned and expressed in *Escherichia coli* (yielding an enzyme designated as rXynT6-E) and in *Pichia pastoris* (yielding rXynT6-P). The recombinant xylanases were found to have optimal activity at 47–55°C and pH 6.0–7.0. The recombinant xylanase expressed in *P*. *pastoris* has 40% higher thermal stability than that expressed in *E*. *coli*. The recombinant xylanases retained 100% of activity after 10 h incubation in the pH range 3–11 and 68% of activity after 1 h at pH 2.0. The xylanase activities of rXynT6-E and rXynT6-P under optimal conditions were 1030.2 and 873.8 U/mg, respectively. The good stability in a wide range of pH and moderate temperatures may make the xylanase from *Bacillus sonorensis* T6 useful for various biotechnological applications, e.g., as an enzyme additive in the feed industry.

## Introduction

Hemicellulose and cellulose are the main heterogeneous polysaccharides of plant biomass [1]. Hemicelluloses consist of residues of various pentoses (d-xylose and d-arabinose) and hexoses (d-mannose, d-glucose, and/or d-galactose) [2]. Hemicelluloses composed of β-d-xylose residues are xylans, which are the main component of hemicellulose. β-1,4-Xylans are heteropolysaccharides with a homopolymeric backbone chain of 1,4-linked β-d-xylopyranose units [3]. Two major hemicellulases responsible for xylan hydrolysis are xylanase (EC 3.2.1.8) and β-xylosidase (EC 3.2.1.37) [2]. Xylanases are endoenzymes that hydrolyze homopolymers to release xylooligomers, and β-glycosidases hydrolyze xylooligomers to xylose [4]. Xylanases are being researched intensively because they are used in the food and feed industries, in the bleaching of pulp and paper, and in textile and biofuel production [5, 6]. Xylanases are produced by many organisms, e.g., bacteria, algae, fungi, protozoa, gastropods, and arthropods [7]. Microbial xylanases are preferred catalysts for xylan hydrolysis owing to their high specificity, mild reaction conditions, negligible loss of activity, and insignificant formation of side products [8]. Microbial xylanases are single-subunit proteins with molecular weights in the range of 8–145 kDa [9]. The optimum temperature for xylanases of fungal and bacterial origin varies between 40°C and 60°C. Bacterial xylanases are generally more thermostable than fungal ones [8]. Some xylanases from extremophilic bacteria have an optimum at 80–105°C [10, 11]. A large number of bacterial and fungal producers of natural xylanases are known [5, 12–18]. Xylanases also are well suitable for cloning and homologous and heterologous expression, and the host strains are *Escherichia coli* [19–23], *Bacillus subtilis* [24], *Lactobacillus plantarum* [25], *Pichia pastoris* [26–31], *Pichia stipites* [32], *Saccharomyces cerevisiae* [33–36], and *Kluyveromyces lactis* [37, 38]. The high potential of xylanases for the processing of plant biomass stimulates a search for new enzymes and development of new technologies.

Xylanases with a post-translational modification are of particular interest. It has been noted that glycosylated xylanases have greater thermal stability and resistance to adverse environmental factors [28, 38, 39]. In this regard, research on the production of xylanases seems promising in yeast, which has a well-developed glycosylation system, abundantly secretes heterologous proteins, and can be cultivated under submerged fermentation conditions in high-capacity bioreactors [29, 30]. The methylotrophic yeast *P*. *pastoris* has proved to be a highly successful system for a variety of recombinant proteins. As a single-celled microorganism, *P*. *pastoris* is easy to manipulate and grows rapidly on inexpensive media at high cell density [40].

The aim of this work was biochemical characterization of xylanase XynT6 from *Bacillus sonorensis* T6. For this purpose, the xylanase was obtained in recombinant form as a nonglycosylated protein in *E*. *coli* (the rXynT6-E enzyme) and as a glycosylated protein in *P*. *pastoris* (the rXynT6-P enzyme). A comparative analysis of the two versions of the enzyme was carried out in terms of temperature and pH optima, thermal and pH stability, and effects of metal ions, detergents, and solvents. Substrate specificity and the degree of xylan hydrolysis were investigated, and kinetic parameters were determined. The suitability of the yeast producer for large-scale fermentation was demonstrated.

## Materials and methods

Animal maintenance and experimental procedures were in accordance with the Kazakhstan national regulations based on the provisions of the Central Ethics Commission of the Ministry of Health of the Republic of Kazakhstan (Nur-Sultan, Kazakhstan) and provisions of the European Convention for the Protection of Vertebrate Animals Used for Experimental and other Scientific Purposes (ETS 123, Strasbourg, 1986).

### Chemicals, microorganisms, plasmids, and primers

Restriction enzymes, DNA polymerases, and T4 DNA ligase were purchased from Thermo Fisher Scientific (USA), and endoglycosidase H from New England Biolabs (USA). The plasmid pJET1.2/Blunt Cloning Kit (Thermo Fisher Scientific) was used for the cloning of the xylanase gene. Plasmids pET28c(+) (Novagen, UK) and pPICZαA (Invitrogen, USA) were employed to construct expression and shuttle vectors. *E*. *coli* strains DH5α and ArcticExpress (DE3) RP were bought from Thermo Fisher Scientific and Novagen (Merck4Biosciences, France), respectively, whereas *P*. *pastoris* X-33 cells from Invitrogen. Birchwood xylan, cellulose, carboxymethyl cellulose, starch (Sigma-Aldrich, St. Louis, MO, USA), beechwood xylan (Megazyme, Ireland), and pullulan (Tokyo Chemical Industry, Tokyo, Japan) served as substrates. The chemical reagents used in this work were of molecular biology or pure analytical grade and purchased from Sigma-Aldrich and AppliChem (Darmstadt, Germany). All primers used in this study are listed in Table 1.

**Table 1:** Primers for cloning and sequencing.

Name	Primers
27F	5′-AGAGTTTGATCCTGGCTCAG-3′
1492R	5′-TACGGTTACCTTGTTACGACTT-3′
Xyn_fw	5′-ATGTTTAAGTTTAAAAAGAATTTCTTAG-3′
Xyn_rv	5′-TTACCACACTGTTACGTTAGAACT-3′
XynT6_NcoI	5′-CATGCCATGGCTAGCCCAGACTACTGGCAA-3′
XynT6_NotI	5′-TTTTCCTTTTGCGGCCGCCCACACTGTTACGTTAGAACTTC-3′
XynT6_EcoRI	5′-CCGGAATTCGCTAGCCCAGACTACTGGC-3′
pJET1.2_F	5′-CGACTCACTATAGGGAGAGCGGC-3′
pJET1.2_R	5′-AAGAACATCGATTTTCCATGGCAG-3′
Т7_fw	5′-TAATACGACTCACTATAGGG-3′
Т7_rv	5′-TAATACGACTCACTATAGGG-3′
AOX1_F	5′-GACTGGTTCCAATTGACAAGC-3′
AOX1_R	5′-GCAAATGGCATTCTGACATCC-3′

### Growth media

Several media were utilized in this work: Lennox Broth (1% of tryptone, 0.5% of yeast extract, 0.5% of NaCl, pH 7.5), super optimal broth with catabolite repression (1% of tryptone, 0.5% of yeast extract, 0.05% of NaCl, 2.5 mM KCl, 20 mM MgSO_4_, 20 mM glucose, pH 7.5), Yeast Extract Peptone (YEP) (1% of yeast extract, 2% of peptone, pH 6.0), YEPD (YEP + 2% of dextrose), YEPG (YEP + 1% of glycerol), YEPM (YEP + 1% of methanol). Media ingredients were acquired from Sigma-Aldrich, AppliChem, and Titan (India). Nutrient Broth (Himedia, India) (1% of peptone, 1% of beef extract, 0.5% of NaCl, pH 7.3) and Basal medium (BM) (0.3% of NaNO_3_, 0.05% of K_2_HPO_4_, 0.02% of MgSO_4_∙7H_2_O, 0.002% of MnSO_4_∙H_2_O, 0.002% of FeSO_4_∙H_2_O, 0.002% of CaCl_2_∙2H_2_O, 0.5% of yeast extract, pH 7.2) were used for the cultivation of *Bacillus* strains.

### Isolation and identification of bacteria

The bacteria were isolated from soil in Southern Kazakhstan. The isolation procedure was as follows: 1 g of soil was mixed with 9 mL of 0.9% (w/v) NaCl, vortexed, and diluted 10-fold; next, 0.1 mL of the mixture was spread on a Nutrient agar plate and incubated at 37°C for 48 h. Colonies were picked, transferred onto Basal agar with 1% of birchwood xylan, and cultivated at 37°C for 48 h. Separate colonies found on the xylan agar plate were identified and screened for xylanase activity.

Taxonomical analyses and morphological examination of the colonies were conducted by light microscopy. The screening of the microorganisms for xylanolytic activity was performed by the Congo red assay [41]. The bacterial isolates were cultured overnight at 37°C in plates containing 1.5% of agar in BM with 1% of birchwood xylan. The agar medium in the culture plates was covered with a 0.1% (w/v) Congo red solution and incubated for 15 min with intermittent shaking. After that, the plates were washed with 1 M NaCl.

Genomic DNA from the cells was isolated with the Monarch Nucleic Acid Purification Kit (New England Biolabs). The gene coding for a small subunit of the ribosome (16S rRNA) was amplified by PCR and sequenced for identification. The amplification was performed with a universal primer pair: 27F and 1492R. PCRs (50 μL final volume) contained 5 μL of 10X Taq Buffer (Thermo Fisher Scientific), 3 μL of 25 mM MgCl_2_, 5 μL of dNTPs (a 2 mM stock solution), 1 μL of each primer (a 10 μM stock solution), 100 ng of a DNA template, 1 μL of Taq DNA polymerase (5000 U/mL), and 34 μL of nuclease-free water. The following amplification parameters were utilized: initial denaturation at 95°C for 5 min; then 30 cycles of 95°C for 1 min, 55°C for 1 min, and 72°C for 1 min; and final extension at 72°C for 10 min. Sequences were compared with GenBank data using the Basic Local Alignment Search Tool (http://blast.ncbi.nlm.nih.gov/Blast.cgi).

Identification by matrix-assisted laser desorption ionization (MALDI) time-of-flight (TOF) mass spectrometry (MS) was performed by means of BiotyperMicroflex LT (Bruker Daltonics, Bremen, Germany).

### SDS-PAGE, zymography, and liquid chromatography coupled with tandem mass spectrometry

*Bacillus* strains were inoculated into three types of medium (BM with 1% of wheat bran, 0.5% birchwood xylan, or 0.5% birchwood xylan with 0.01% of xylose) and cultured at 37°C and 120 rpm in a shacking incubator for 120 h. The supernatant was clarified by centrifugation (10 000 × *g*, 30 min, 4°C) and subjected to SDS-PAGE in a 10% polyacrylamide gel copolymerized with 0.2% (w/v) of birchwood xylan without β-mercaptoethanol. The gel was washed for 1 h in 2.5% Triton X-100 and then incubated in 100 mМ phosphate buffer (pH 6.8) at 50°C for 1 h according to Vandooren [42]. The gel was stained with 0.1% Congo red and destained in 1 M NaCl for 10 min. The decolorization band was excised from the gel and subjected to nano-high-performance liquid chromatography quadrupole time-of-flight mass-spectrometry. The proteins were extracted from the gel and trypsinized. An unmodified CaptiveSpray ion source was employed to interface the liquid-chromatography system with an Impact II mass spectrometer (Bruker, Germany). The Mascot software was used to perform searches against the NCBInr 20140923 database (49,710,996 sequences; 17,838,311,419 residues), taxonomy—Bacteria (Eubacteria) (33,609,539 sequences).

### Expression of xylanase in *E*. *coli* and purification of rXynT6-E

The xylanase gene was amplified from bacterial genomic DNA with primers Xyn_fw and Xyn_rv and cloned into the pGET1.2/blunt vector according to the manufacturer’s instructions. Ampicillin-resistant (Amp^R^) clones were found by PCR screening for the insertion with primers pJET1.2_F and pJET1.2_R. Plasmids from three PCR-positive clones were extracted by means of the GeneJET Plasmid Miniprep Kit (Thermo Fisher Scientific) and the inserts were sequenced.

The *xynT6* gene was amplified from plasmid pJET1.2/xynT6 with PCR primers XynT6_NcoI and XynT6_NotI and was cloned into the pET28c(+) vector at NcoI and NotI sites, resulting in expression plasmid pET28/xynT6. The correctness of the insertion was confirmed by sequencing of the *T7* locus with primers Т7_fw and Т7_rv. *E*. *coli* ArcticExpress(DE3)RP cells were electroporated with pET28/xynT6 and the resulting kanamycin-resistant (Kn^R^) transformants were cultured in 500 mL of Lennox Broth with kanamycin (50 μg/mL) at 37°C and 150 rpm. Isopropyl β-d-1-thiogalactopyranoside (IPTG; 0.5 mM) was added when the culture reached 600 nm optical density of 0.6, and the culture was incubated for 16 h. The cells were collected by centrifugation (6000 × *g*, 7 min, 4°C), resuspended in 20 mM Tris-HCl (pH 8.0) with 500 mM NaCl and lysed with lysozyme (2 mg/mL) followed by ultrasonication. The lysate was clarified by centrifugation (40 000 × *g*, 1 h, 4°C) and passed though a 0.45 μm filter. The rXynT6-E protein was isolated from the cell lysate by metal affinity chromatography on an AKTA Purifier 10 FPLC chromatograph (GE Healthcare, USA) using a 1 mL HiTrap Chelating column (GE Healthcare) activated with Ni^2+^ and equilibrated with a buffer consisting of 20 mM imidazole, 500 mM NaCl, and 20 mM Tris-HCl (pH 8.0). The rXynT6-E protein was eluted via a 20–500 mM imidazole gradient in 20 mM Tris-HCl (pH 8.0) with 500 mM NaCl. Protein purity was verified by SDS-PAGE.

### Anti-rXynT6-E antibody preparation and western blotting

A 6-month-old rabbit was immunized five times with the rXynT6-E protein (0.15–0.30 mg) during 35 days. Two days later, the sixth immunization was carried out, and the next day, blood serum was collected and the presence of an anti-rXynT6-E antibody was checked by immunoblotting. Western blotting was performed according to the standard protocol [43]. Briefly, proteins in samples were separated by SDS-PAGE in a 12% (w/v) gel and transferred to a polyvinylidene difluoride membrane. The membrane was blocked with 5% (w/v) solution of skim milk powder in Tris-buffered saline supplemented with Tween 20 (TBST: 50 mM Tris-HCl pH 7.6, 150 mM NaCl, 0.1% [w/v] of Tween 20). The xylanase of *Bacillus sonorensis* T6 was detected by means of the rabbit polyclonal antibody against rXynT6-E (at 1:1000 dilution) as a primary antibody and a goat anti-rabbit IgG antibody conjugated with horseradish peroxidase at 1:10 000 dilution as a secondary antibody. The bands were detected with the Enhanced Chemi-Luminescence Detection Kit (AppliChem), and an X-ray film (AgfaPhoto GmbH, Germany) was then exposed to the membrane.

### Expression of xylanase in *P*. *pastoris* and purification of rXynT6-P

The *xynT6* gene was amplified with primers XynT6_EcoRI and XynT6_NotI and was cloned into the pPICZαA vector, resulting in shuttle plasmid pPICZα/xynT6. The pPICZα/xynT6 plasmid was linearized with endonuclease PmeI. *P*. *pastoris* X-33 cells were electroporated with the purified linearized plasmid. The resultant zeocin-resistant (Zeo^R^) transformants were screened by PCR with primers AOX1_F and AOX1_R. PCR-positive clones were tested for xylanase activity. Cells of a clone with the highest activity were inoculated into 20 mL of YEPG and were cultured for 24 h at 30°C and 250 rpm. The cells were harvested by centrifugation (3,500 × *g*, 15 min, 4°C), the pellet was resuspended in YEPM, and the culture was incubated at 30°C and 220 rpm for 120 h. The cells were collected and discarded. The supernatant (80 mL) was treated with ammonium sulfate (60% saturated solution) and stored at 4°C for 16 h. After that, it was centrifuged (40 000 × *g*, 60 min, 4°C), and the pellet was dissolved in 4 mL of a buffer composed of 200 mM sodium phosphate and 100 mМ citric acid (pH 6.0) and dialyzed against the same buffer using a 14 kDa molecular weight cutoff membrane. The dialyzed solution was loaded onto a column with Sephadex G-100 (Sigma-Aldrich). The column was washed with the above buffer at a flow rate of 0.01 mL/min by means of AKTA Purifier 10 FPLC, the collected fractions were tested for xylanase activity, and the protein concentration was measured.

### Glycosylation prediction and validation

The glycosylation of rXynT6-P was predicted in NetNGlyc 1.0 and NetOGlyc 4.0 on the basis of the amino acid sequence. Moreover, the r-XynT6-P glycosylation was experimentally verified by western blotting using the purified protein with or without pretreatment with endoglycosidase Endo H. Concentration of purified XynT6-P was adjusted to 1 mg/mL, and XynT6-P was denatured at 95°C for 5 min. Deglycosylation was performed by endoglycosidase H (New England Biolabs) at 37°C for 16 h. The reaction was stopped by heating at 65°C. The result was analyzed by western blotting.

### Measurement of protein concentration and a xylanase assay

Protein concentration was determined by the Bradford method (Protein Assay Dye; Bio-Rad, Munich, Germany) with bovine serum albumin as the standard. Briefly, 100 μL of the Bradford reagent was mixed with 860 μL of 10% PBS with 1% of glycerol and added 40 μL of a protein sample. The mixture was incubated for 2 min at room temperature (RT), and optical density was measured on a spectrophotometer at 595 nm. The measurements were performed on three independent replicates, and the average of the three samples is reported. Xylanase activity was determined by the 3,5-dinitrosalicylic acid method [17]. In brief, birchwood xylan was dissolved at 1% concentration (w/v) in 100 mM phosphate buffer (pH 7.0 for rXynT6-E and 6.0 for rXynT6-P) and used as a substrate. Next, 1 mL of the substrate was incubated with 40 μL of a diluted enzyme at 50°C for 15 min. The reaction was stopped by the addition of 1.5 mL of 3,5-dinitrosalicylic acid reagent, and the mixture was boiled in a water bath for 10 min. Absorbance was measured at 540 nm. A calibration curve was constructed using xylose (Thermo Fisher Scientific [Acros Organic, USA]) as a standard. One unit of xylanase activity is defined as the amount of the enzyme that liberates 1 μmol of reducing sugar (xylose) per minute under the standard conditions (50°C, pH 6.0 or 7.0). All measurements were performed three times independently, and the average of three replicates was reported as the defined result.

### Effects of temperature and pH on xylanase activity and stability

The xylanase activity was measured across a temperature range of 10–80°C (with 5°C intervals) in 100 mM phosphate buffer with pH 7.0 or 6.0 for rXynT6-E and rXynT6-P, respectively. Maximum enzymatic activity was regarded as 100% activity, and the other samples at different temperatures were assayed for relative activity accordingly. To determine the impact of temperature on enzyme stability, an xylanases were preincubated at 40°C, 55°C, 60°C, or 70°C in an optimal buffer for 1–4 h. Initial activity was set to 100%, and residual activity was calculated relative to the initial activity.

The enzymatic activity was measured in the pH range of 2.0 to 10.0 (with half-unit intervals) at optimal temperature. Maximum enzymatic activity was regarded as 100% activity, and the other samples at different pH were assayed for relative activity accordingly. To determine the effect of pH on enzyme stability, xylanases were preincubated at RT in buffers having different pH levels (3–11 with 2-unit intervals) for 10 h and at pH 1.5 and 2.0 for 90 min. The enzymatic activity was measured at optimal temperature and pH. Initial activity was set to 100%, and residual activity was calculated relative to the initial activity. The following buffer systems were used: KCl-HCl (pH 1.5) glycine-HCl (pH 2.0–3.5), sodium acetate buffer (pH 4.0–5.5), phosphate buffer (pH 6.0–8.5), and glycine-NaOH (pH 9.0–12.0).

### Effects of metal ions, detergents, and other chemicals on enzyme stability

The influence of metal ions on xylanase stability was examined by assaying rXynT6-E and rXynT6-P in the presence of one of eight chlorides—NiCl_2_, MgCl_2_, CaCl_2_, CuCl_2_, ZnCl_2_, MnCl_2_, FeCl_3_, and CoCl_2_—at 5 mM. The enzyme was preincubated with metal chloride at RT for 1 h, and then the activity was measured under the conditions optimal for each enzyme. The following detergents and chemicals were tested for their influence on xylanase activity of rXynT6-E and rXynT6-P: Triton X-100 (0.5% v/v), ammonium sulfate (100 mM), sodium dodecyl sulfate (SDS; 10 mM), urea (100 mM), β-mercaptoethanol (0.5% v/v), guanidine hydrochloride (100 mM), dithiothreitol (10 mM), EDTA (10 mM), methanol (5%), ethanol (5%), isopropyl alcohol (5%), and acetone (5%). Enzymatic activity without preincubation with metal ions, detergents, and other chemicals served as a control and was set to 100%.

### Determination of kinetics and substrate specificity

The kinetic analysis of rXynT6-E and rXynT6-P was performed after a 15 min reaction with 1–15 mg/mL birchwood or beechwood xylan as a substrate under optimum reaction conditions. K_m_ and V_max_ were calculated via the Michaelis–Menten equation [44]. Substrate specificity of rXynT6-E and rXynT6-P was determined under optimal reaction conditions in the presence of 1% (v/v) of starch, cellulose, carboxymethyl cellulose, pullulan, birchwood, or beechwood xylan.

### Scanning Electron Microscopy (SEM)

Wheat straw samples were treated with 18% ammonia and incubated with either rXynT6-E or rXynT6-P for 6 h at 50°C and for 72 h at 37°C in phosphate buffer. A sample incubated under the same conditions without xylanases served as a control. To avoid contamination with microorganisms, sodium azide (2%, w/v) was added to all samples. After treatment, the samples were analyzed by SEM for morphological changes. The samples were placed on stubs and gold-sputtered (10 nm). Images were captured on an Auriga Crossbeam 540 (Carl Zeiss, Germany) scanning electron microscope operating at 3 kV.

### Enzyme production in a bioreactor

A 10 L fermenter (Biostat, Sartorius, Germany) was utilized to determine the ability of each yeast strain to produce recombinant xylanase XynT6-P on a large scale. A single colony was inoculated into 5 mL of YEPD broth and grown at 30°C and 250 rpm. After 24 h, the culture was transferred to 30 mL of YEPD broth and grown under the same conditions for 24 h. The culture was inoculated into 300 mL of YEPG broth and grown under the same conditions for 24 h. Then, the culture was inoculated into 1 L of YEPG broth and grown under the same conditions for 24 h. After that, the cells were collected by centrifugation (3000 × *g*, 5 min, RT), and the pellet was resuspended in 5 L of YEPM broth in the 10 L bioreactor. Standard procedures were followed to operate the fermenter under the following cultivation conditions: 30°C, 300–400 rpm, aeration 2–4 L/min, pH 6.0, a fed carbon source: 1% of methanol (added each 24 h), and cultivation duration: 144 h. Wet cell weight and xylanase activity of the supernatant were measured during the cultivation.

### Software tools and bioinformatic analysis

All measurements were performed three times independently. Means and standard deviations were calculated in the GraphPad Prism software, version 8.0.1. Enzymatic activities are presented as means, and other parameters as the mean ± standard deviation (n  =  3). Calculation of a protein’s molecular weight and isoelectric point, nucleotide sequencing, primer design, and other manipulations were performed using Vector NTI Advance 11 and SnapGene Viewer 5.2.4 software. Nucleotide and protein sequences were compared with the NCBI nucleotide/protein database by means of BLASTN and BLASTP online programs, respectively. Online software Peptide Signal IP 5.0 (http://www.cbs.dtu.dk/services/SignalP/) was used for predicting a signal peptide region. Glycosylation sites were predicted in NetNGlyc 1.0 (http://www.cbs.dtu.dk/services/NetNGlyc/) and NetOGlyc 4.0 Server (http://www.cbs.dtu.dk/services/NetOGlyc/).

## Results and discussion

### Isolation and identification of a xylanolytic strain

**Fig 1:**
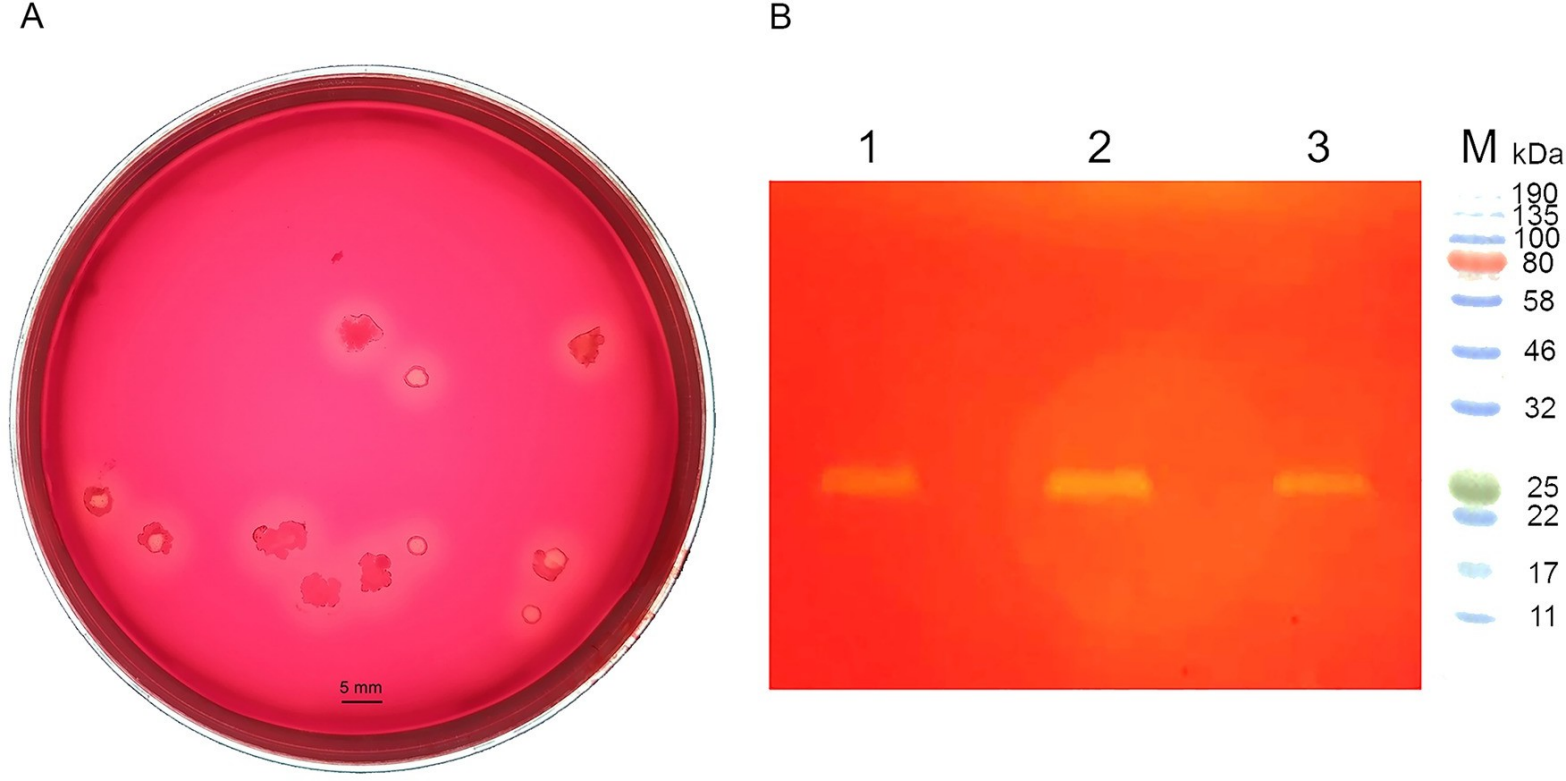
Colonies of *Bacillus sonorensis* T6 on a xylan agar plate stained with Congo red (A) and zymographic analysis of the *Bacillus sonorensis* T6 culture supernatant (B). The strain was cultivated on BM with 1% of wheat bran (lane 1), 1% of birchwood xylan (lane 2), 1% of birchwood xylan, or 0.01% of xylose (lane 3); protein molecular-weight markers: Color Protein Standards, Broad Range (New England Biolabs, cat. # P7712S) (lane M).

Eight bacterial strains were isolated from a Sothern Kazakhstan soil. After 24 h on nutrient agar, the bacteria produced beige colonies 1–2 mm in diameter with wavy edges. The cells turned out to be gram-positive, oval, mobile, and forming spores. By sequencing and comparing the fragment of 16S rRNA gene nucleotide sequence with GenBank data and Biotyper data, the strains were identified as *Bacillus sonorensis*. Screening showed that only the *Bacillus sonorensis* T6 strain has xylanolytic activity. *Bacillus sonorensis* T6 displayed xylanolytic activity by forming a white halo with an average diameter of 2.3 mm around the colonies (Fig 1A). Zymographic analysis revealed that the *Bacillus sonorensis* T6 strain secretes an extracellular enzyme (Fig 1B) with a molecular weight of ~23 kDa.

The proteins of the clear zone from the zymographic analysis were trypsinized, and the peptides in the mixture were separated by nano-high-performance liquid chromatography. The peptides were electrospray-ionized, parental and daughter ions were obtained, and their spectra were determined on a quadrupole time-of-flight mass spectrometer. Bioinformatic analysis of the spectra on the Mascot platform identified peptides belonging to the endo-1,4-β-xylanase of *Bacillus sonorensis* or uncultured bacteria with scores 304 and 101, respectively (Table 2). The endo-1,4-β-xylanase is affiliated with glycosyl hydrolases [45] and is often found in bacteria of the genus *Bacillus* [12, 14–16, 18–21, 23, 26, 46, 47].

**Table 2:** Results of Mascot searches in the NCBInr database by means of liquid chromatography–tandem mass spectrometry data.

NCBInr ID	Protein	Molecular mass (M_r_)	Peptide	Total sequence coverage (%)	Total score
gi|493688735	endo-1,4-beta-xylanase [*Bacillus sonorensis*]	23308	GTVYSDGGTYDIYTTTR	12	304
			TTFTQYWSVR		
gi|115338505	beta-1,4-endoxylanase [uncultured bacterium]	23309	SDGGTYDIYTTTR	10	101
			TTFTQYWSVR		

Primers were chosen based on xylanase sequences from GenBank for such *Bacillus* species as *B*. *sonorensis*, *B*. *subtilis*, *B*. *licheniformis*, *B*. *paralicheniformis*, *B*. *amyloliquefaciens*, *B*. *cereus*, *B*. *halotolerans*, *B*. *inaquosorum*, and *B*. *velezensis* (accession No. MK774668, CP053102, MF288581, CP005965, CP041693, MN339588, MT121978, MT121976, and MT121975, respectively). The *xynT6* gene was amplified, cloned into the pJET1.2/blunt vector, and sequenced. Analysis of the amino acid sequence of the transcript of *xynT6* confirmed that xylanase XynT6 belongs to glycosyl hydrolase family 11. The first 28 amino acid residues (MFKFKKNFLVGVTAALMSISVFSATASA) of the XynT6 sequence were identified as a signal peptide. The nucleotide sequence of the *xynT6* gene from *Bacillus sonorensis* T6 was deposited in GenBank (accession No. MZ733680).

### Expression and purification of rXynT6-E

**Fig 2:**
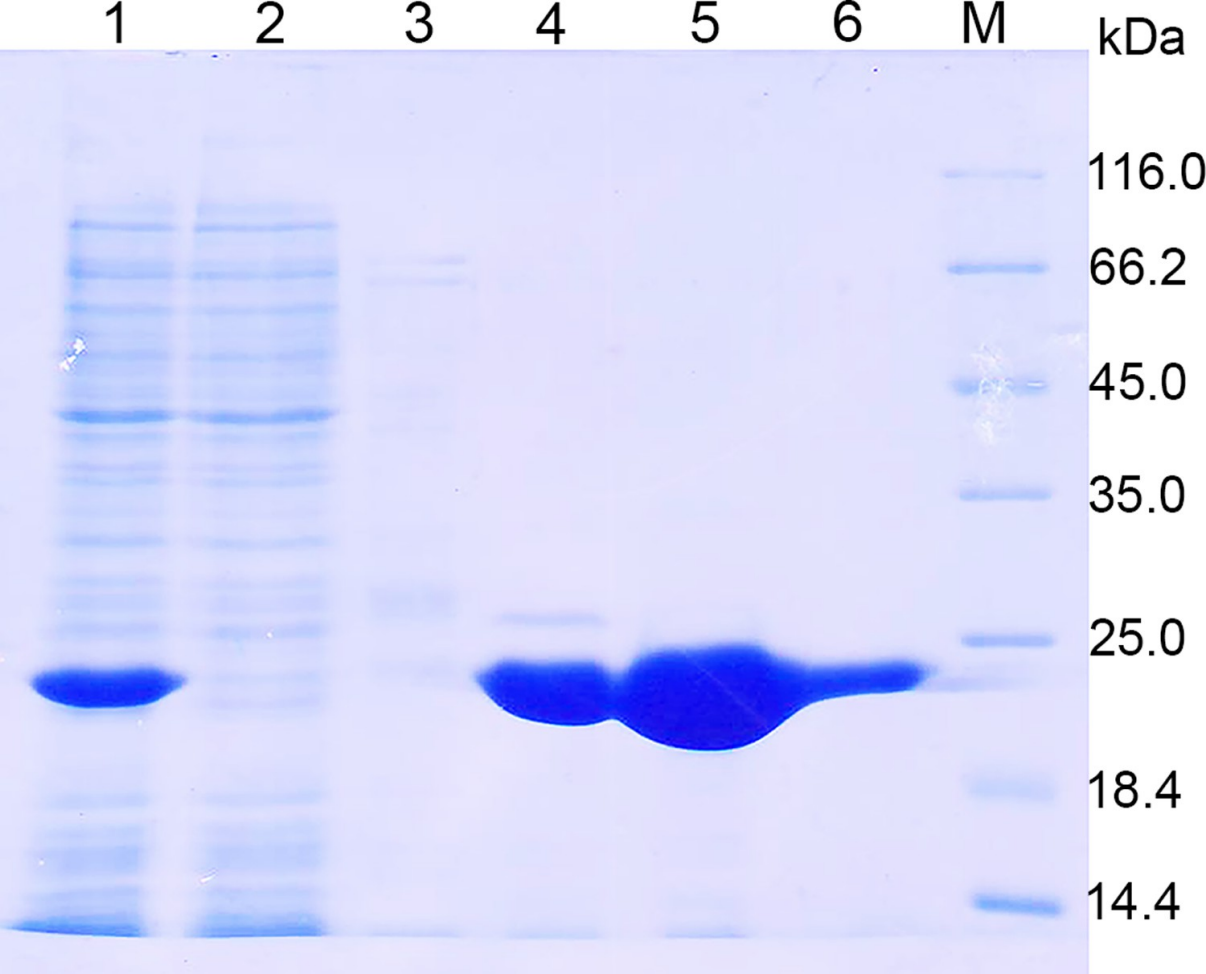
SDS-PAGE results on rXynT6-E purification. A clarified lysate (supernatant) of IPTG-stimulated *E*. *coli* ArcticExpress (DE3)RP_pET-28/XynT6 cells (lane 1), the supernatant passed through column with Ni^2+^ (lane 2), fractions eluted with 20 mM imidazole (lane 3) or 232 mM imidazole (lanes 4–6), and Unstained Protein Molecular Weight Markers (Thermo Fisher Scientific, cat. # 26610) (lane M).

The *xynT6* gene without the signal peptide was cloned into vector pET28(+) for expression in *E*. *coli* with a hexahistidine tag. The calculated molecular weight of recombinant XynT6-E with the 6×His tag is 28.3 kDa. By transformation of strain ArcticExpress (DE3), strain RP_pET-28/XynT6 was obtained, which efficiently produced the rXynT6-E xylanase. The accumulation of the recombinant xylanase was noted at 3 h after the addition of IPTG, and rXynT6-E was mostly in a water-soluble form. The rXynT6-E protein was purified by Ni^2+^ affinity chromatography and eluted from the column at 232 mM imidazole (Fig 2).

SDS-PAGE analysis showed high purity of rXynT6-E (Fig 2). The xylanase assay suggested that after the purification, the loss of enzymatic activity was less than 10% (Table 3). Thus, rXynT6-E was successfully purified and found to be concentrated 8.5-fold. The specific activity of the purified enzyme was 939.1 U/mg.

**Table 3:** Purification of recombinant xylanases rXynT6-E and rXynT6-P from *E*. *coli* ArcticExpress (DE3)RP_pET-28/XynT6 and *P*. *pastoris* X-33_pPICZα/XynT6.

Purification step	Xylanase activity, U	Total protein, mg	Specific activity, U/mg	fold enrichment	Recovery, %
rXynT6-E					
Clarified supernatant	6090	55.2	110	1	100
Ni^2+^ affinity chromatography	1174	1.8	939.1	8.5	27.8
rXynT6-P					
Culture supernatant	11880	50	237.6	1	100
(NH_4_)_2_SO_4_ precipitation and dialysis	7630	12	635.8	2.7	64.2
Sephadex G-100	499.5	0.6	832.5	3.5	4.2

### Expression and purification of N-glycosylated rXynT6-P

The *xynT6* gene was cloned into the pPICZαA vector under the *AOX1* promoter. In the vector, the proprietary signal peptide of xylanase was replaced with the α-MF peptide from *S*. *cerevisiae* for secretion of the resulting rXynT6-P protein from yeast cells. The calculated molecular weight of the recombinant XynT6-P xylanase is 29.9 kDa. By transformation of X-33 cells, clones were obtained that produced rXynT6-P after the addition of 1% of methanol as a source of carbon. Table 4 shows the results of screening of culture supernatants of six clones for xylanase activity.

**Table 4:** Results of screening of culture supernatants of *P*. *pastoris* X-33_pPICZα/XynT6.

Clone	Xylanase activity, U/mL	Clone	Xylanase activity, U/mL
1	110.6	4	91.3
2	108.3	5	98
3	114.5	6	96
Control	0		

**Fig 3:**
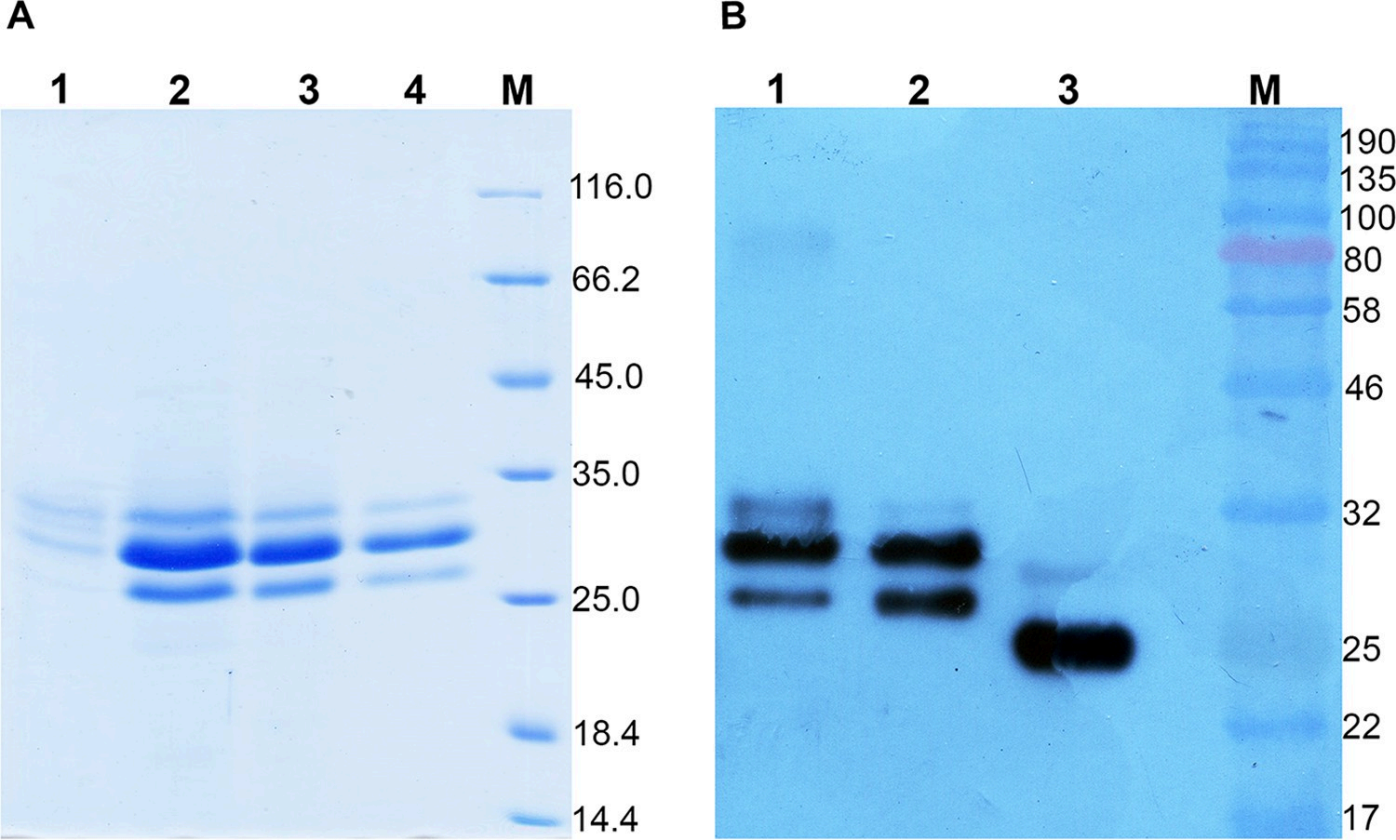
SDS-PAGE analysis of rXynT6-P purification (A) and western blotting analysis (B). **(A)** The culture supernatant of *P*. *pastoris* X-33_pPICZαA/XynT6 (lane 1), the ammonium sulfate precipitate (lane 2), a fraction after dialysis (lane 3), a fraction after Sephadex G-100 chromatography (lane 4), and Unstained Protein Molecular Weight Markers (Thermo Fisher Scientific, cat. # 26610) (lane M). (**B**) The culture supernatant of strain *P*. *pastoris* X-33_pPICZα/XynT6 (lane 1), purified rXynT6-P (lane 2), and purified rXynT6-P treated with EndoH (lane 3). The protein markers: Color Protein Standards, Broad Range (New England Biolabs, cat. # P7712S).

Clone # 3 (114.5 U/mL) has the highest activity and was chosen as a yeast producer of rXynT6-P. Testing confirmed the stability of the *xynT6* insert in the genome of the recombinant strain. Purification of rXynT6-P by precipitation with ammonium sulfate followed by purification by size exclusion chromatography on Sephadex G-100 was effective (Fig 3A). The rXynT6-P protein precipitated from the supernatant at 80% (NH₄)₂SO₄ concentration, and the loss after dialysis was 35.8% (Table 3). Size exclusion chromatography was performed to remove any remaining components of the culture media and ammonium sulfate. Thus, rXynT6-P was purified successfully and concentrated 3.5-fold. The specific activity of purified rXynT6-P was 832.5 U/mg. Analysis of its amino acid sequence suggested that XynT6-P contains three potential sites of N-glycosylation: Asp53-Tyr54-Ser55, Asp57-Trp58-Ser59, and Asp209-Val210-Thr211. Treatment of rXynT6 with endoglycosidase Endo H confirmed that rXynT6-P is highly glycosylated, as evidenced by three bands on a western blot before glycosylation and two bands after treatment with Endo H (Fig 3B). The addition of mannose chains raises the molecular weight of the rXynT6-P protein by approximately 5.0–5.5 kDa above the predicted value. Glycosylation can ensure correct protein folding, prevent proteolytic degradation, and facilitate intracellular transport [48]. Moreover, glycosylation is one of the greatest advantages of the yeast *P*. *pastoris* as a protein producer [40]. Glycosylation has been documented for xylanases from *Thermobifida fusca* and *Streptomyces* sp. S38 [28, 31].

### Effects of pH and temperature on xylanase activity and stability

**Fig 4:**
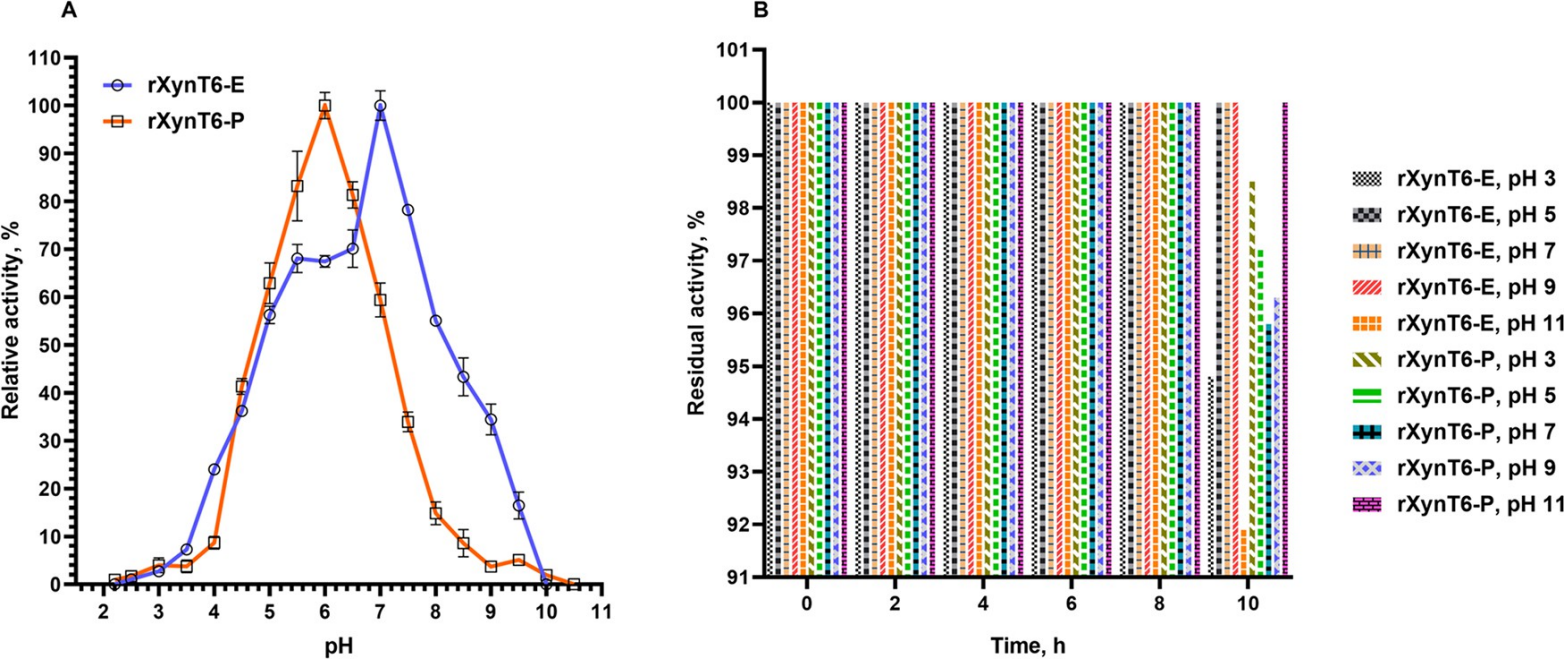
The influence of pH on the activity (A) and stability (B) of rXynT6-E and rXynT6-P.

A comparative analysis of the two recombinant xylanases suggested that the pH optimum of activity of the glycosylated xylanase (rXynT6-P) is lower than that of the nonglycosylated xylanase (rXynT6-E) (Fig 4A). The optimum pH for rXynT6-E and rXynT6-P proved to be 7.0 and 6.0, respectively, in agreement with data on bacterial xylanases (Table 5). rXynT6-E retained most of activity (55%) at pH 8.0, whereas rXynT6-P (expressed in the yeast) retained only 14% of activity at the same pH. At pH values below 4.0 and above 10, both enzymes lost activity. pH stability of rXynT6-E and rXynT6-P was investigated by preincubation of the enzymes in buffers with pH 3.0–11.0 at RT for 10 h (Fig 4B). Both enzymes showed high tolerance of both acidic and alkaline conditions. In the pH range 3–11, both enzymes retained 100% activity. Glycosylation did not influence pH stability of the XynT6 enzyme. There are data in the literature on pH stability of xylanases; the enzymes that have been studied to date retain no more than 80% of activity after incubation for 1 h in the pH range 5.0–9.6 [20, 21, 26].

**Table 5:** A comparison of biochemical properties between various bacterial xylanases and rXynT6-E and rXynT6-P.

Enzyme	Strain	MW, kDa	pH optimum	Temperature optimum, °C	Specific activity, U/mg	Reference
rXynT6-E	*Bacillus sonorensis* T6	28.3	7	55	1030.2	Present study
rXynT6-P	*Bacillus* sonorensis T6	35.4	6	47	873.8	Present study
Xyn11	*B*. *licheniformis* MS5-14	23.4	5–7	40–50	-	[19]
Xyn10A	*Bacillus* sp. SN5	45.0	7	40	105	[20]
Bpu XynA	*B*. *pumilus* ARA	23.3	6.6	50	62.8	[21]
Xylanase	*B*. *licheniformis* DM5	38	6.5	50	-	[18]
Xylanase X-II	*B*. *licheniformis* 77–2	17	7	75	367	[16]
Xylanase X-I	*B*. *licheniformis* ALK-1	46	7–9	50	206	[46]
Xylanase	*B*. *amyloliquefaciens* SK-3	50	7–9	50	217.4	[12]

**Fig 5:**
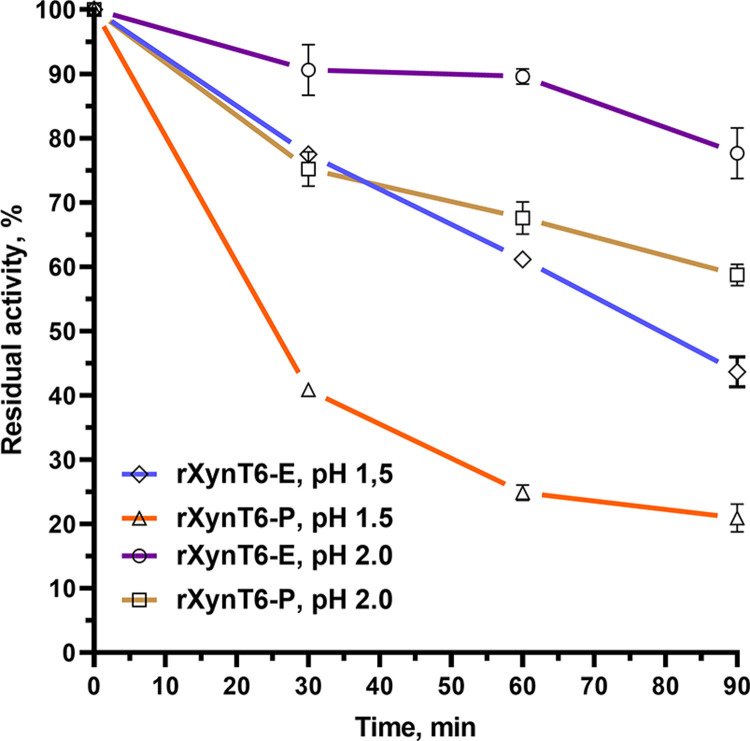
The effect of pH on the stability of rXynT6-E and rXynT6-P in buffers with pH 1.5 and 2.0.

Preincubation of rXynT6-E and rXynT6-P at pH 1.5 or 2.0 for 90 min revealed that rXynT6-E retains more than 80% and more than 90% of activity after preincubation for 30 min, whereas rXynT6-P retains 40% and 70% of activity at pH 1.5 and 2.0, respectively (Fig 5). The pH stability results showed that XynT6 is highly stable under both acidic and alkaline conditions.

The pH stability data are fascinating when considering the exoenzymes used as feed additives for monogastric species like birds, pigs, and fish. Exoenzymes must withstand severe gut conditions, including sudden changes in pH. The chicken gastrointestinal tract is characterized by different pH values in different sections: the crop (pH 5.0–6.0), proventriculus (1.5–4.0), gizzard (2.0–5.0), duodenum (6.0–8.0), small intestine (6.0–7.5), and colon (7.0) [49–53]. Enzymes vary in the optimum pH range under which they can effectively break down substrate material. Enzymes that have a pH range between 3.0 and 7.0 can function within more sections of the digestive tract. The chicken digestive tract is short, taking approximately 4.5 h from ingestion to elimination. The high pH stability of the XynT6 xylanase makes it a promising candidate for a feed exoenzyme.

**Fig 6:**
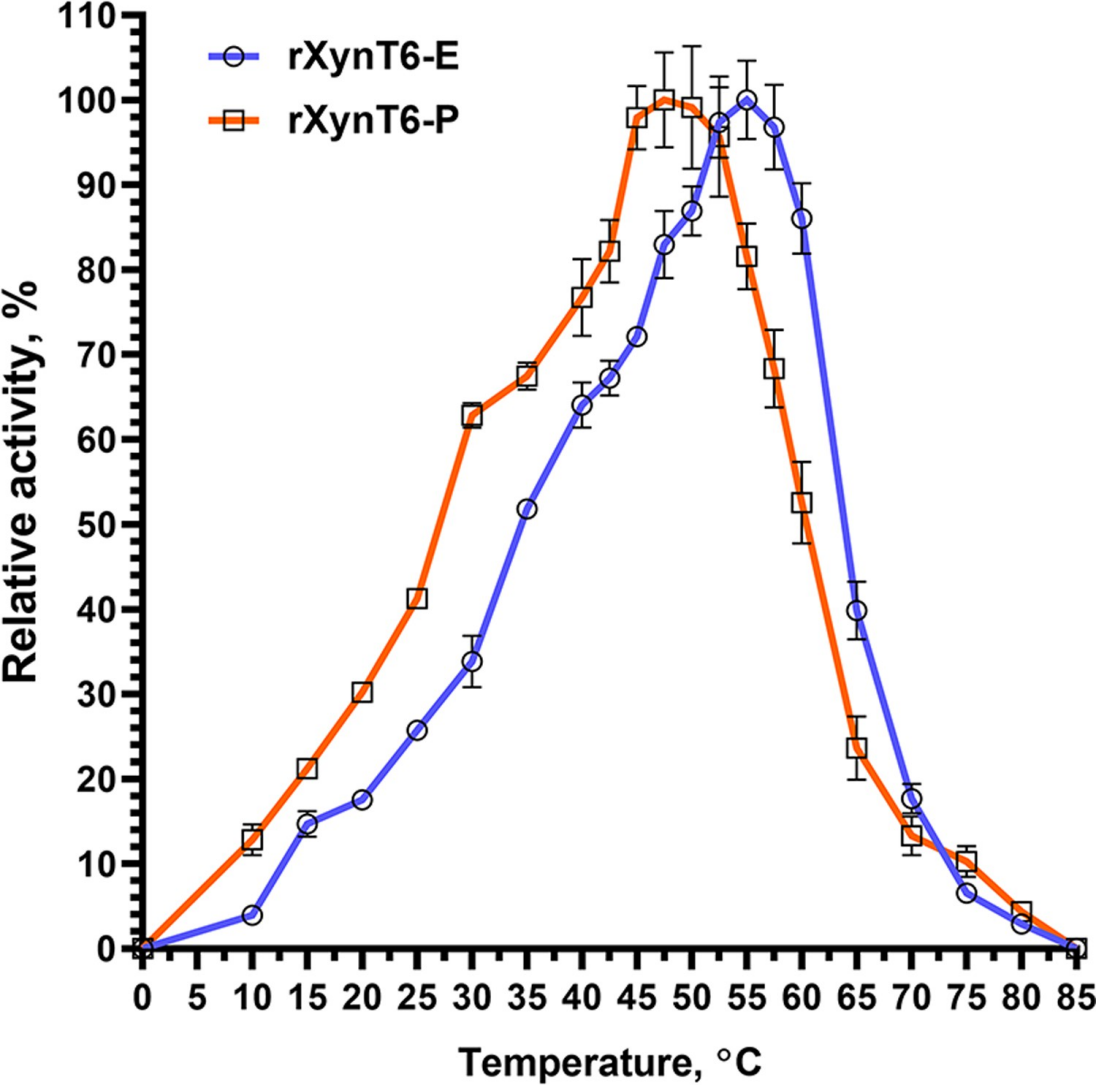
The effect of temperature on the activity of rXynT6-E and rXynT6-P.

Determination of xylanase activity of rXynT6-E and rXynT6-P at various temperatures (10–80°C) suggested that the temperature optima are different between the two enzymes. The optima for rXynT6-E and rXynT6-P are 55°C and 47°C, respectively (Fig 6). At 55°C, rXynT6-P retained more than 80% of activity, and at 60°C, rXynT6-E showed 86% of activity. Above 80°C, both enzymes got completely inactivated. Because these two enzymes are identical in amino acid sequence, the difference in optimum temperature appears to be related to N-glycosylation. A comparative analysis suggested that most of bacterial xylanases have an optimum of 40–50°C (Table 5) [12, 15, 18–21, 27, 41, 54]. More thermostable xylanases from *Bacillus subtilis* ASH and *Bacillus* sp. SV-34S have an optimum at 55°С, just as rXynT6-E does [22, 55].

The maximum activity of rXynT6-E and rXynT6-P toward birchwood xylan under the optimum conditions was 1030.2 and 873.8 U/mg: higher than that of known xylanases (Table 5).

**Fig 7:**
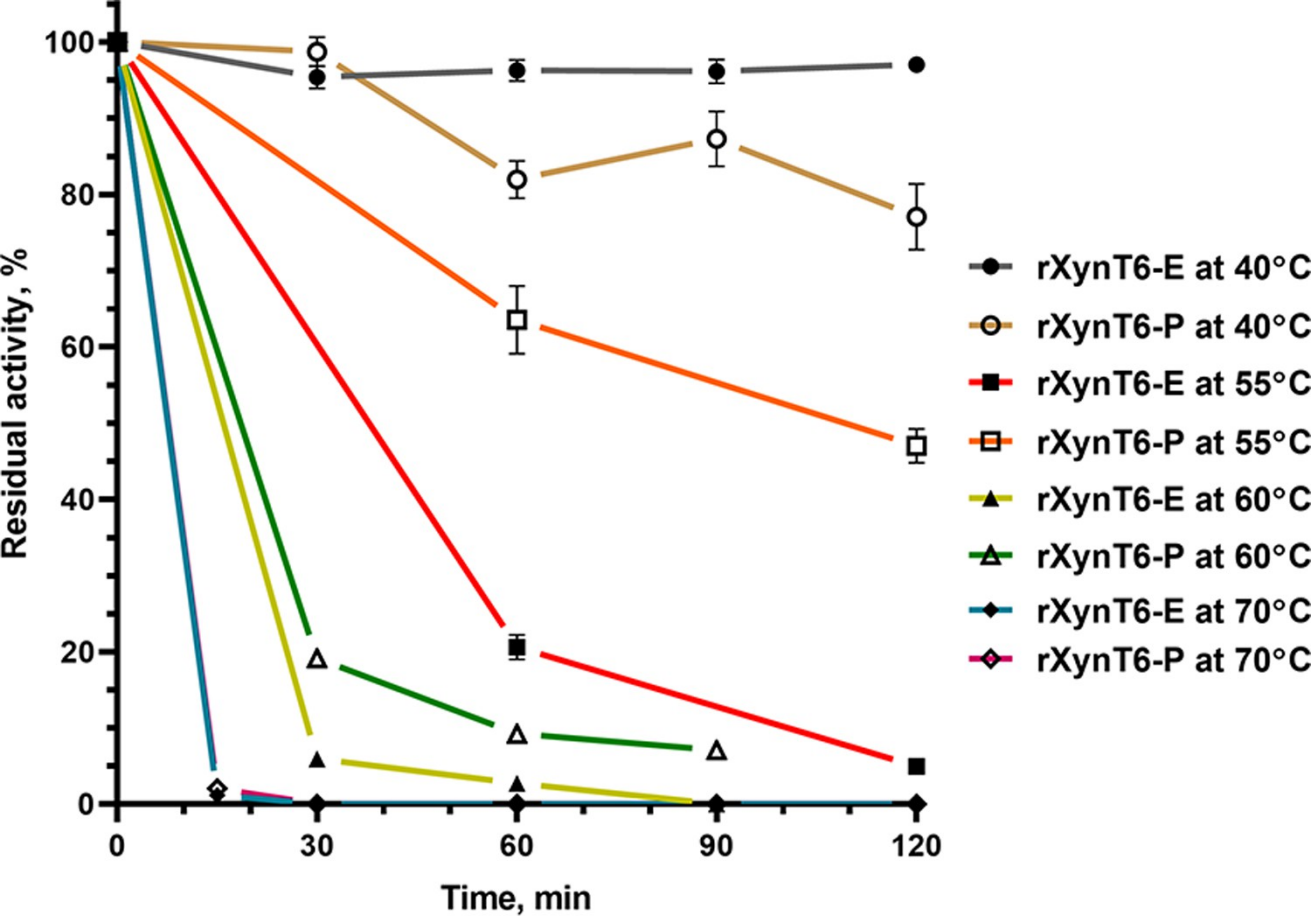
The impact of temperatures 40°C, 55°C, 60°C, 70°C on the stability of rXynT6-E and rXynT6-P.

Thermal stability was investigated by preincubation of the enzymes at 40°C, 55°C, 60°C, or 70°C (Fig 7) at optimal pH for 120 min. The preincubation of rXynT6-E and rXynT6-P at 40°C did not reduce the activity of both enzymes below 80%. Although the XynT6 xylanase obtained in *E*. *coli* has a higher temperature optimum than its N-glycosylated analog obtained in yeast, rXynT6-P possesses higher thermal stability (Fig 7). rXynT6-P retained 47% of activity after preincubation at 55°C for 120 min, whereas rXynT6-E retained no more than 5% of activity. The increase in thermal stability is related to the presence of mannose chains in rXynT6-P, which stabilize its tertiary structure. After preincubation at 60°С for 30 min, rXynT6-P and rXynT6-E had only 19.2% and 5.9% of activity, respectively, and preincubation at 70°C for 30 min completely inactivated both enzymes.

### The influence of inhibitors, reducing agents, metal ions, and organic solvents on xylanase stability

Table 6 presents the effects of metal ions Ni^2+^, Mg^2+^, Ca^2+^, Zn^2+^, Mn^2+^, Cu^2+^, Fe^3+^, and Co^2+^ on the stability of rXynT6-E and rXynT6-P. Ni^2+^, Mg^2+^, Ca^2+^, Zn^2+^, and Fe^3+^ (separately) inhibited xylanase activity of rXynT6-E by 8–26%. The activity of rXynT6-E increased by 18% after preincubation with Mn^2+^. Mg^2+^, Ca^2+^, or Mn^2+^ increased the activity of rXynT6-P by 5–22%. On the contrary, Mn^2+^ has an inhibitory effect on xylanases from *B*. *licheniformis* MS5-14 [19], *Bacillus* sp. SN5 [20], and *B*. *stearothermophilus* T-6 [13]. rXynT6-P was inhibited by 7–42% by Co^2+^, Ni^2+^, and Fe^3+^. Copper ions inhibited rXynT6-E activity almost twofold (57% of residual activity), consistently with the reports on other xylanases [19–22], but rXynT6-P was found to be resistant to the action of Cu^2+^ (90% of residual activity). This is especially interesting because all xylanases show high sensitivity to copper ions [12, 13, 19–22, 46]. Preincubation of rXynT6-E and rXynT6-P with 0.5% Triton X-100 increased xylanase activity of both enzymes: by 17% and 4%, respectively. SDS at 10 mM strongly suppressed the enzymatic activity, by more than 70%; by contrast, 0.5% β-mercaptoethanol and 10 mM DTT increased the activity by 4–20%. rXynT6-E and rXynT6-P manifested good tolerance to urea and guanidine hydrochloride. Preincubation of rXynT6-P with urea increased the activity by 4%, and preincubation with guanidine hydrochloride increased the activity of rXynT6-E by 21%. EDTA at 10 mM increased the activity of rXynT6-E by 16%. Organic solvents exerted different effects on the activity of rXynT6-E and rXynT6-P. Preincubation with acetone or ethanol decreased the activity of rXynT6-E by 5–7%, whereas methanol and isopropanol increased the activity by 3%. For rXynT6-P, only ethanol exerted a stimulatory action: by 16%; methanol and isopropanol reduced the enzymatic activity by 10–20%, and acetone did not affect the activity of rXynT6-P (Table 6).

**Table 6:** Effects of metal cations, detergents, organic solvents, and other chemicals on xylanase activity of rXynT6-E and rXynT6-P.

Chemicals	Concentration	Residual activity, %	
		rXynT6-E	rXynT6-P
None	-	100 ± 0.9	100 ± 4.3
Ni^2+^	5 mM	73.8 ± 1.3	96.3 ± 4.4
Mg^2+^	5 mM	92.1 ± 2.3	116.7 ± 4.1
Ca^2+^	5 mM	92.3 ± 3.3	105.7 ± 1.8
Zn^2+^	5 mM	83.5 ± 0.5	99.5 ± 2.4
Mn^2+^	5 mM	118.2 ± 4.6	122.6 ± 3.9
Co^2+^	5 mM	101.6 ± 0.5	92.5 ± 2.8
Cu^2+^	5 mM	56.9 ± 3.5	90.0 ± 1.2
Fe^3+^	5 mM	78.4 ± 0.9	58.1 ± 3.9
Triton X-100 (v/v)	0.5%	117 ± 0.9	104.1 ± 1.0
SDS	10 mM	28.4 ± 1.1	24.4 ± 0.6
β-Mercaptoethanol (v/v)	0.5%	120.9 ± 1.5	118.9 ± 3.2
Dithiothreitol	10 mM	104.7 ± 0.2	107.6 ± 5.1
Urea	100 mM	101 ± 0.9	95.7 ± 2.9
Guanidine hydrochloride	100 mM	121.2 ± 1.2	102.6 ± 0.4
EDTA	10 mM	116.4 ± 4.6	102.2 ± 17.2
Methanol	5%	102.8 ± 13.8	80.7 ± 2.8
Ethanol	5%	92.9 ± 7.9	116.8 ± 8.2
Isopropyl alcohol	5%	103.4 ± 4.6	90.0 ± 9.2
Acetone	5%	95.8 ± 8.3	99.5 ± 1.5

The values are averages from three independent assays.

### Substrate specificity and determination of K_m_, V_max_, and K_cat_

rXynT6-E and rXynT6-P manifested relatively good substrate specificity: 100% and 100% relative activity toward birchwood xylan and 113.4% and 117.6% relative activity toward beechwood xylan, respectively. Almost no activity was detected when the substrate was cellulose, carboxymethyl cellulose, starch, or pullulan, indicating high specificity and the absence of cellulase, amylase, and pullulanase activities in XynT6. Similar substrate specificity has been reported for xylanases from *Bacillus* sp. SN5 and *Nesterenkonia xinjiangensis* CCTCC AA001025 [20, 22].

K_m_, V_max_, and K_cat_ of the recombinant enzymes were determined using different concentrations of birchwood or beechwood xylan as a substrate (Table 7). A comparison of the K_m_ values toward these xylans suggested that the affinity of rXynT6-E and rXynT6-P is higher for birchwood xylan than for beechwood xylan.

**Table 7:** Kinetic parameters of rXynT6-E and rXynT6-P.

Parameter	Values			
	rXynT6-E		rXynT6-P	
Substrate	Birchwood xylan	Beechwood xylan	Birchwood xylan	Beechwood xylan
Specific activity (U/mg)	939.1 ± 20.8	1065.3 ± 16.2	832.5 ± 22.7	978.7 ± 9.22
K_m_ (mg/mL)	2.973 ± 0.393	6.779 ± 0.819	3.037 ± 0.362	4.965 ± 0.434
V_max_ (U/mg)	987.5 ± 53.6	1691.9 ± 101.5	667.8 ± 31	355.8 ± 13.7
K_cat_ (s^-1^)	148.3 ± 8.1	254 ± 15.1	100.3 ± 4.6	53.42 ± 2.5

### SEM analysis of the pulp treated with rXynT6-P

**Fig 8:**
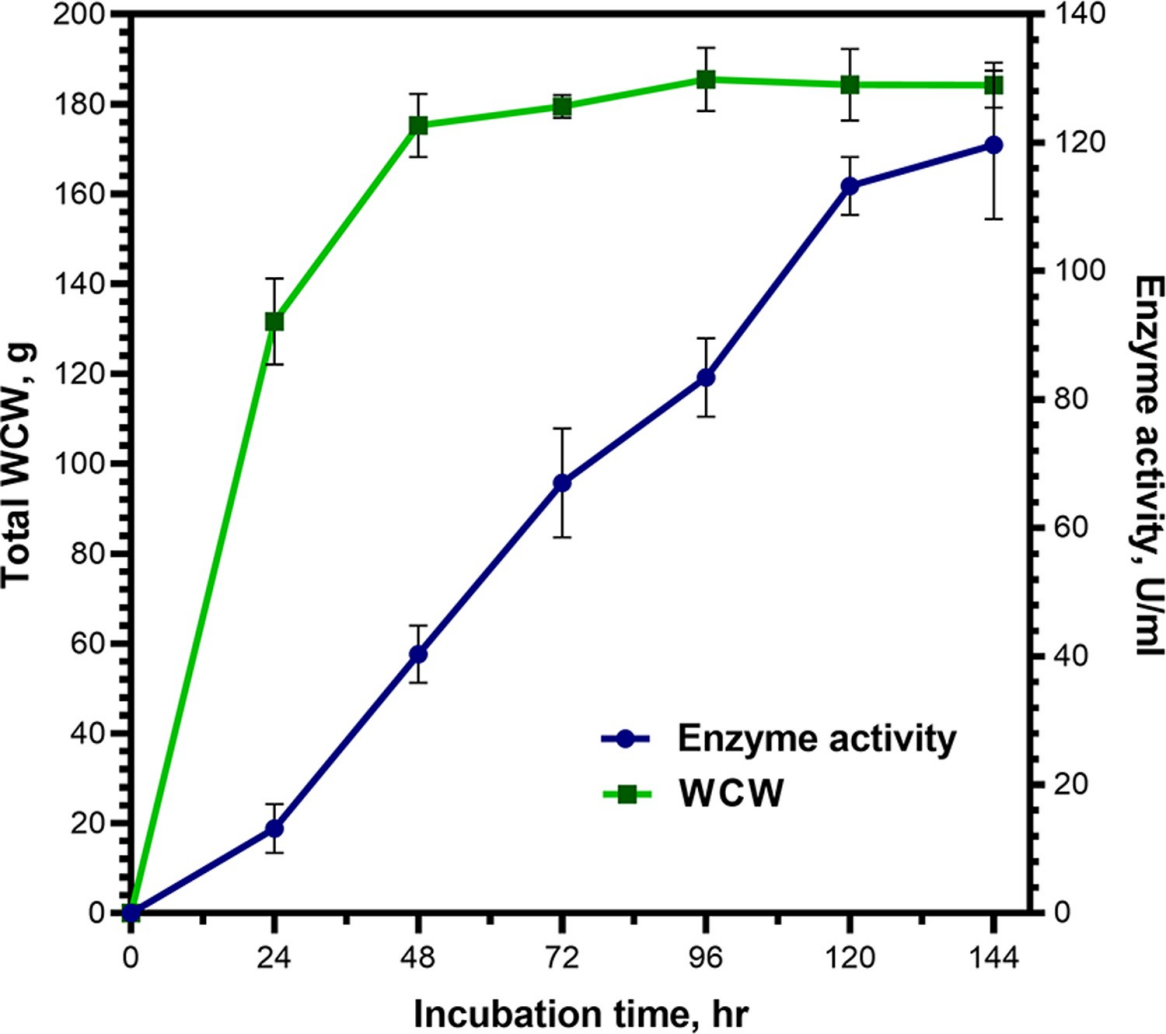
SEM images of the untreated (A) and rXynT6-E–treated (B) or rXynT6-P–treated (C) wheat straw at ×1000 magnification.

SEM analysis of wheat straw samples was performed next to examine morphological changes after treatment with rXynT6-E or rXynT6-P. SEM images of untreated and treated samples (Fig 8) uncovered morphological changes after the treatment with recombinant xylanases. The surface of untreated wheat straw was smooth (Fig 8A) (without any roughness). By contrast, the surface of wheat straw after treatment was rough (Fig 8B and 8C). The samples of treated wheat straw showed irregularities, roughness areas suggestive of degradation, which means the hydrolysis of xylan.

### Production of rXynT6-P in a pilot bioreactor

**Fig 9:**
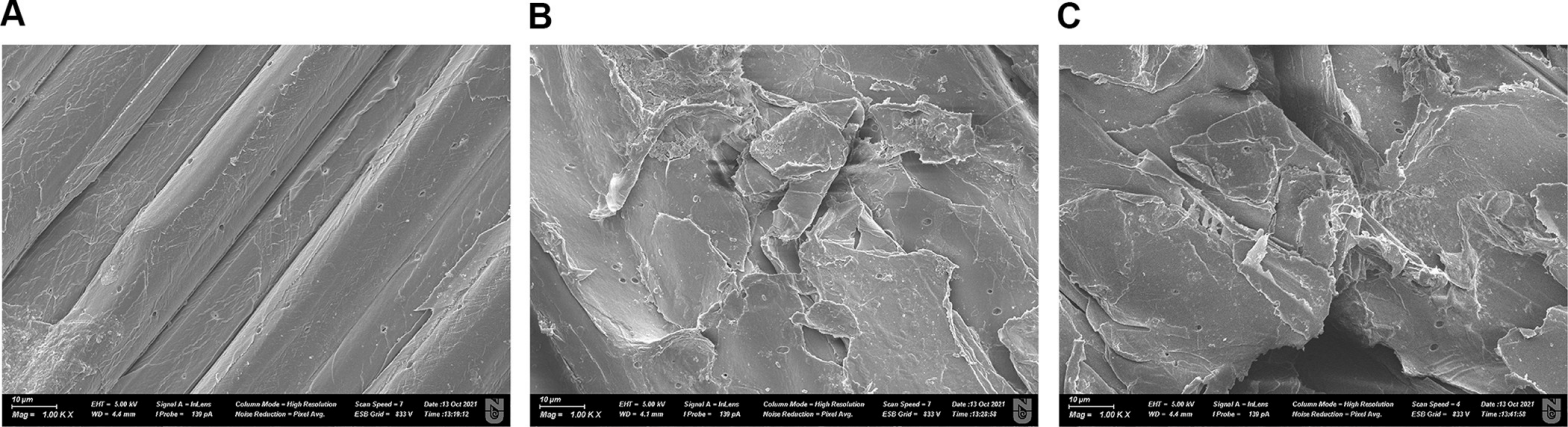
Pilot scale production of the recombinant xylanase by the yeast *P*. *pastoris*. WCW: wet cell weight, U: Units.

The 10 L bioreactor was employed to culture the *P*. *pastoris* X-33/pPICZα/XynT6 yeast strain to investigate the possibility of large-scale production of the recombinant xylanase (Fig 9). A wet cell weight of 47.4 g/L was obtained after 144 h growth. The resultant supernatant contained 120 U/mL activity at the end of the fermentation. The total yield of the recombinant enzyme was 2529.7 U per gram of wet cells. The yield of native xylanase XynT6 when *Bacillus sonorensis* T6 was cultured on BM for 72 h was 0.4 U/mL, which is 167 times less than that of the recombinant *P*. *pastoris* X-33/pPICZα/XynT6 strain for the same cultivation period (67 U/mL). For other known natural producers of xylanase, the yield is 1.47–10.00 U/mL [13–15], which is significantly lower than that of the recombinant producer *P*. *pastoris* X-33/pPICZα/XynT6. These results indicate that XynT6 expression in *P*. *pastoris* under the control of the methanol-inducible promoter is effective.

## Conclusion

A xylanase gene of the *Bacillus sonorensis* T6 strain was cloned and expressed in *E*. *coli* (yielding the rXynT6-E enzyme) and *P*. *pastoris* (yielding rXynT6-P). The recombinant xylanases have excellent stability in the pH 1.5–11.0 range. rXynT6-P featuring N-glycosylation manifested good thermal stability at 55°C and maximum activity at 47°C. Preparation of rXynT6-P in a 10 L fermenter indicates that the recombinant yeast in question may be suitable for large-scale production of xylanase. The high resistance to acidic and alkaline pH and good thermal stability of the N-glycosylated xylanase imply good potential for a wide variety of applications in the biotech industry.

## Supporting information

S1 Raw images Raw images of gels and blot. These raw images of SDS-PAGE results of purification for rXynT6-E (Fig 2) and SDS-PAGE analysis of rXynT6-P purification and western blotting analysis (Fig 3).
(PDF)
