# Supplementary material for: Cloning, expression, and characterization of a recombinant xylanase from Bacillus sonorensis T6
Source: PLoS One. 2022 Mar 17;17(3):e0265647. doi: 10.1371/journal.pone.0265647 (PMC8929556; doi:10.1371/journal.pone.0265647)
Supplement: S1 Raw images — These raw images of SDS-PAGE results of purification for rXynT6-E (Fig 2) and SDS-PAGE analysis of rXynT6-P purification and western blotting analysis (Fig 3). (PDF) [file pone.0265647.s001.pdf]

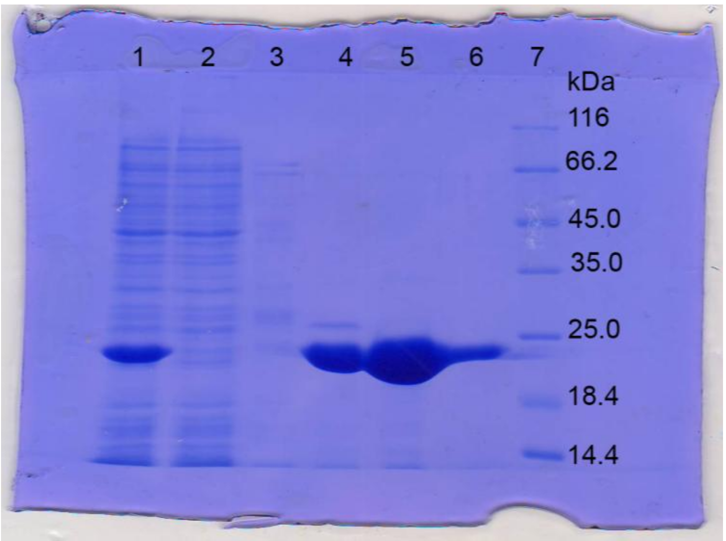

Fig 2. SDS-PAGE results on rXynT6-E purification. A clarified lysate (supernatant) of IPTG-stimulated *E. coli* ArcticExpress (DE3)RP_pET-28/XynT6 cells (lane 1), the supernatant passed through column with Ni^{2+} (lane 2), fractions eluted with 20 mM imidazole (lane 3) or 232 mM imidazole (lanes 4–6), and Unstained Protein Molecular Weight Markers (Thermo Fisher Scientific, cat. # 26610) (lane M).

A

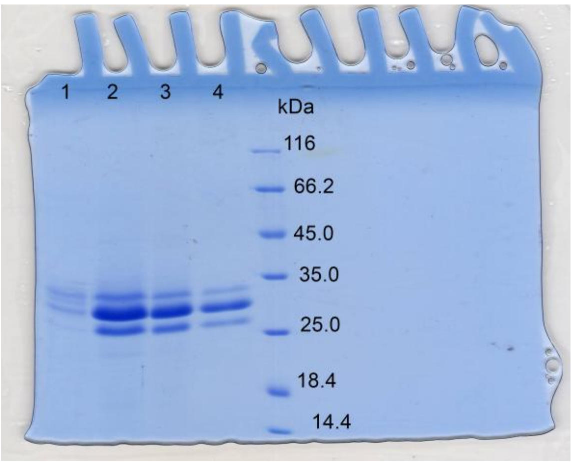

B

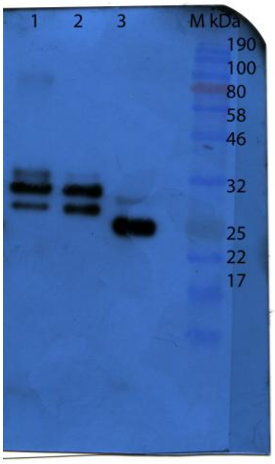

Fig 3. SDS-PAGE analysis of rXynT6-P purification (A) and western blotting analysis (B). **(A)** The culture supernatant of *P. pastoris* X-33_pPICZαA/XynT6 (lane 1), the ammonium sulfate precipitate (lane 2), a fraction after dialysis (lane 3), a fraction after Sephadex G-100 chromatography (lane 4), and Unstained Protein Molecular Weight Markers (Thermo Fisher

Scientific, cat. # 26610) (lane M). (**B**) The culture supernatant of strain *P. pastoris* X-33_pPICZα/XynT6 (lane 1), purified rXynT6-P (lane 2), and purified rXynT6-P treated with EndoH (lane 3). The protein markers: Color Protein Standards, Broad Range (New England Biolabs, cat. # P7712S).
